# Potential association between endocrine disrupting chemicals (EDCs) and gynecomastia: a systematic review based on partial experimental evidenceendocrine-disrupting chemicals

**DOI:** 10.3389/fendo.2026.1734644

**Published:** 2026-02-03

**Authors:** Haobo Wang, Mengdie Wang, Cuihong Fan, Yueyao Sun, Jianping Feng, Meng He, Ning Li, Fangjian Shang, Bo Liu

**Affiliations:** The First Hospital of Hebei Medical University, Shijiazhuang, Hebei, China

**Keywords:** endocrine disrupting chemicals (EDCs), exposure risks, gynecomastia, hormonal imbalance, mechanism of action

## Abstract

Gynecomastia (GYN), the most prevalent benign breast condition in men, is primarily driven by an estrogen-androgen imbalance, which induces glandular proliferation and adipose hypertrophy. This imbalance leads to the proliferation of mammary gland tissue and hypertrophy of adipose tissue. Endocrine-disrupting chemicals (EDCs), as a class of exogenous substances widely distributed in the environment, can disrupt hormonal homeostasis by mimicking estrogen, antagonizing androgens, or interfering with hormone metabolism. Consequently, they represent a significant environmental risk factor for inducing male breast development. Some evidence also suggests that gynecomastia may represent a chronic, non-infectious inflammatory response to estrogenic stimulation, which could further alter the tissue’s hormonal sensitivity. This paper systematically reviews typical EDCs associated with male breast development, including bisphenols, phthalates, and polycyclic aromatic hydrocarbons. It examines their exposure pathways and mechanisms of action, analyzes the clinical characteristics, public health implications, and current prevention and control status of gynecomastia. The review highlights existing issues in current research, such as unclear mechanisms and the complexity of mixed exposure effects. It proposes that future efforts should focus on strengthening research into the molecular mechanisms linking EDCs to male breast development, improving population exposure monitoring systems, and refining prevention and control strategies. This will provide a theoretical basis for the scientific prevention and clinical management of the condition. Furthermore, the paper emphasizes the critical importance of strictly controlling the environmental release of EDCs to protect public health.

## 1 Introduction

Gynecomastia (GYN), the most prevalent male breast disorder, accounts for approximately 85% of male breast pathologies (1). It clinically presents as unilateral or bilateral breast enlargement, typically featuring a palpable, firm, and tender subareolar mass. The pathogenesis of GYN, whether physiological, pathological, drug-induced, or idiopathic, is fundamentally linked to an imbalance in the estrogen-to-androgen ratio—particularly the estradiol-to-testosterone ratio (E2/T) (2). Excessive levels of estrogen or its precursors in males, increased sensitivity of estrogen receptors, androgen deficiency, androgen receptor resistance, or an abnormal estrogen-to-androgen ratio within normal hormone ranges may all contribute to male breast development. Since R. Marcus and S. G. Korenman first established the link between gynecomastia and estrogen levels in 1976 (3), subsequent research has consistently confirmed that hormonal imbalance is the core driving factor in the pathogenesis of the disease (4). Based on this theory, any pathological state or medication that increases circulating estrogen or reduces circulating androgen will elevate the estrogen-to-androgen ratio, thereby inducing gynecomastia.

Endocrine-disrupting chemicals (EDCs) are exogenous substances or mixtures that interfere with hormonal processes, thereby posing significant risks to human health. By disrupting the endocrine system, EDCs can contribute to various diseases and disorders affecting the reproductive, nervous, and immune systems—including male breast development. In 2012, the World Health Organization (WHO) and the United Nations Environment Programme (UNEP) recognized EDCs as a global public health concern (5). These compounds remain prevalent in numerous consumer and industrial products, such as plastics, pesticides, food packaging, cosmetics, detergents, pharmaceuticals, and electronic components (6). Due to their widespread presence, human exposure to EDCs is virtually unavoidable. Such exposure has been linked to adverse health outcomes, including reproductive abnormalities, metabolic disorders, endocrine dysfunction, gynecomastia, breast cancer, and prostate cancer (7).

Although studies have confirmed associations between certain EDCs and male breast development, the precise mechanisms of action, the “cocktail effect” of mixed exposures, and the susceptibility of vulnerable populations (such as fetuses and adolescents) require further investigation. This paper aims to systematically review the pathways and mechanisms through which EDCs influence male breast development, synthesize evidence from clinical observations and epidemiological studies, analyze the clinical and public health implications of this condition, and provide guidance for future research and prevention efforts.

## 2 Materials and methods

A systematic literature search was conducted using the PubMed database with the following keywords: “endocrine-disrupting chemicals”, “gynecomastia”, “estrogen”, “androgen”, “hormone imbalance”, and “exposure risk”. The search included all publication years.

Inclusion Criteria:

1. Studies investigating the association between EDC exposure and male breast development;
2. Mechanistic research on the effects of EDCs on estrogen-androgen balance;
3. Studies on the clinical characteristics, etiology, and public health implications of male breast development;
4. Epidemiological surveillance data on EDC exposure.

Exclusion Criteria:

1. Studies involving female subjects;
2. Purely toxicological research that fails to establish a clear association between EDCs and male breast development;
3. Duplicated publications or studies of low quality.

Data extraction and meta-analysis were performed on the included literature, focusing on categorizing EDC types, exposure pathways, mechanisms of action, and public health risks.

## 3 Clinical and public health significance of male breast development

### 3.1 Symptom burden and psychosocial impact

The lifetime prevalence of gynecomastia is as high as 65% (8). The manifestation of abnormal breast enlargement contradicts traditional male gender characteristics, often imposing severe psychological and social pressures on affected individuals. This impact is particularly pronounced among adolescent males, potentially disrupting their normal psychological development (9). Research confirms that adolescent gynecomastia is frequently accompanied by multiple psychological issues, including emotional and cognitive disorders such as depression, anxiety, social phobia, and suicidal ideation. It may also trigger behavioral abnormalities such as social withdrawal, academic disruption, eating disorders, and aggressive behavior, forming a vicious cycle of “appearance-related inferiority – social withdrawal – identity disturbance” that severely impairs mental health and social functioning (10).

### 3.2 Early warning signs of potential illnesses

Male breast development is not an isolated breast condition, but often an external manifestation of hormonal imbalance or organ dysfunction within the body, potentially concealing various serious underlying diseases.

#### 3.2.1 Endocrine disorders

Multiple endocrine dysfunctions can induce male breast development by disrupting the estrogen-androgen balance. Hyperthyroidism (Graves’ disease) reduces free testosterone by increasing sex hormone-binding globulin (SHBG), with clinically evident male breast development occurring in 10%-40% of patients (11). Primary gonadal insufficiency (e.g., due to testicular injury, chemotherapy, mumps orchitis, or leprosy) may induce male breast development by lowering serum testosterone levels, elevating luteinizing hormone (LH), and stimulating residual interstitial cells to secrete estrogen (12). Klinefelter syndrome (47,XXY karyotype), a chromosomal disorder, is often associated with hypogonadism and infertility. Approximately 70% of affected males develop gynecomastia, though the precise mechanism remains unclear (12). Male pseudohermaphroditism (Morris syndrome, i.e., testicular feminization) arises from persistent gonadal estrogen secretion, typically presenting with a feminized breast appearance. Hormone testing aids in differential diagnosis: primary hypogonadism is characterized by reduced testosterone and elevated LH, while secondary testicular failure shows decreased testosterone with normal LH levels (13). Additionally, males with long-standing type 1 diabetes may develop diabetic mammary disease, presenting as unilateral or bilateral diffuse, indurated breast enlargement. Histopathology reveals inflammatory changes with lymphocytic infiltration around mammary ducts and lobules (14). Other endocrine and metabolic abnormalities, such as metabolic syndrome, refeeding after severe starvation, significant weight loss, and functional hyperprolactinemia, are also associated with male breast development (15).

#### 3.2.2 Liver and kidney diseases

The liver serves as the primary organ for inactivating estrogen. In patients with liver cirrhosis, impaired hepatic function leads to estrogen accumulation, with 30–60% developing breast enlargement. The degree of enlargement correlates positively with the severity of liver dysfunction (14). Patients with chronic renal failure typically exhibit hypogonadism, characterized by defective testosterone production, which may result in secondary gynaecomastia (15).

#### 3.2.3 Malignant tumors

Various tumors can directly or indirectly induce male breast development by disrupting the body’s hormonal balance, with their mechanisms closely linked to tumor type and secretory characteristics. Germ cell tumors such as testicular tumors and choriocarcinoma increase estrogen levels, disrupting the androgen-estrogen equilibrium and thereby triggering male breast development (12, 13, 15). Pituitary adenomas (prolactinomas) may also induce male breast development by secreting excessive prolactin (16). Adrenal cortical tumors, predominantly observed in young to middle-aged males, can directly secrete steroid precursors such as estrogen and androstenedione; elevated serum estrogen levels further inhibit LH-mediated testosterone synthesis, exacerbating hormonal imbalance and inducing male breast development (12). Additionally, ectopic tumors in organs such as the lungs, liver, stomach, or kidneys may cause male breast development if they secrete human chorionic gonadotropin (hCG) (16, 17).

### 3.3 Widespread exposure to EDCs and population risks

With the widespread use of various industrial products in daily life, exposure pathways to EDCs have permeated all aspects of daily existence. Research has shown that intake of bisphenol compounds is associated with the occurrence of gynecomastia in males (18). Although no direct research evidence exists for other types of EDCs, they may induce male breast development through similar mechanisms, characterized by pervasive and cumulative exposure patterns. EDCs are widely present in products such as plastics (bisphenol A, phthalates), cosmetics (lavender essential oil, tea tree oil), pesticides (atrazine, DDT), and food packaging (polychlorinated biphenyls). Humans are continuously exposed through respiration, diet, and skin contact, and there is virtually no “zero-exposure” population (19). BPA metabolites are detectable in the urine of over 90% of the population (20).

EDCs disrupt hormonal equilibrium by mimicking estrogen (e.g., bisphenol A binding to ERα receptors), antagonizing androgens (e.g., nonylphenol inhibiting testosterone synthesis), or interfering with the hypothalamic-pituitary-gonadal axis (e.g., atrazine inhibiting 5α-reductase). More dangerously, they exhibit a “cocktail effect”—the combined toxicity of multiple EDCs far exceeds that of any single substance (21). The fetal and adolescent stages are sensitive windows for EDC exposure, during which developing breast tissue is vulnerable to hormonal disruption, with effects potentially transmitted across generations. Animal studies indicate that perinatal BPA exposure induces abnormal proliferation of mammary epithelial cells in male offspring, providing insights into male gynecomastia research (18).

### 3.4 Prevention and control gaps resulting from inadequate awareness

Public awareness of gynecomastia remains low, with insufficient attention paid to male breast development. Many patients delay seeking medical attention due to the misconception that “male breast enlargement is not a disease”. Furthermore, primary care facilities often lack sufficient knowledge regarding EDC-related breast development, making it difficult to accurately distinguish between true and false breast enlargement. Some patients are misdiagnosed as having “fat accumulation” and do not undergo further examination, thereby missing opportunities for early detection of underlying conditions such as hyperthyroidism or liver cirrhosis. Moreover, no public health monitoring system currently exists for male breast development. Screening for populations exposed to EDCs and early intervention for obesity-related risks are entirely absent. Screening for breast development in obese men is not included in routine physical examinations, resulting in many early-stage patients going undetected.

The significance of male breast development fundamentally reflects the interaction between “individual health issues” and “environmental-societal factors”. Clinically, it serves as a “warning sign” for endocrine, hepatic, renal, and oncological disorders, requiring precise diagnosis and etiological treatment to improve patient quality of life. From a public health perspective, it reflects societal challenges such as environmental EDC contamination, obesity epidemics, and inadequate health awareness. Mitigating population-level risks requires source control (e.g., restricting EDC use), population interventions (e.g., obesity management), and health education (e.g., adolescent risk awareness). Future efforts should promote synergy between clinical practice and public health surveillance. For example, incorporating EDC exposure monitoring into routine examinations for patients with breast development disorders, and including breast ultrasound screening in health check-ups for obese and elderly males. This will ultimately facilitate a shift from “disease treatment” to “risk prevention”, thereby reducing the long-term impact on population health.

## 4 Pathophysiological basis of male breast development

Male breast development refers to abnormal breast enlargement caused by benign proliferation of mammary gland tissue, which can occur unilaterally or bilaterally. A survey of 50 adolescent males with gynecomastia found that 68% presented with bilateral involvement, while 32% exhibited unilateral cases (22). Particularly during adolescence, this condition may be accompanied by significant distress, psychological pressure, physical anxiety, and social isolation (9). Kinsella et al. diagnosed 24 young males with adjustment disorder (79.2%), anxiety disorder (16.7%), dysthymia (16.7%), and social phobia (4.2%) (9).

Clinically, patients with true gynecomastia often present with palpable nodular or cord-like tissue beneath the areola, which is elastic or moderately firm, frequently accompanied by tenderness or nipple sensitivity (16). In contrast, pseudogynecomastia primarily manifests as increased breast volume without glandular tissue proliferation, with palpation typically revealing soft fatty tissue (23). Histological examination further clarifies the diagnosis: true gynecomastia is characterized by proliferation of mammary duct epithelial cells and increased periductal fibrous connective tissue, with alveoli typically sparse or absent; pseudogynecomastia lacks glandular components and consists solely of fat deposition (24). These distinctions are not only important for clinical differential diagnosis but also provide evidence for investigating the pathophysiological basis of male breast development.

**Figure 1:**
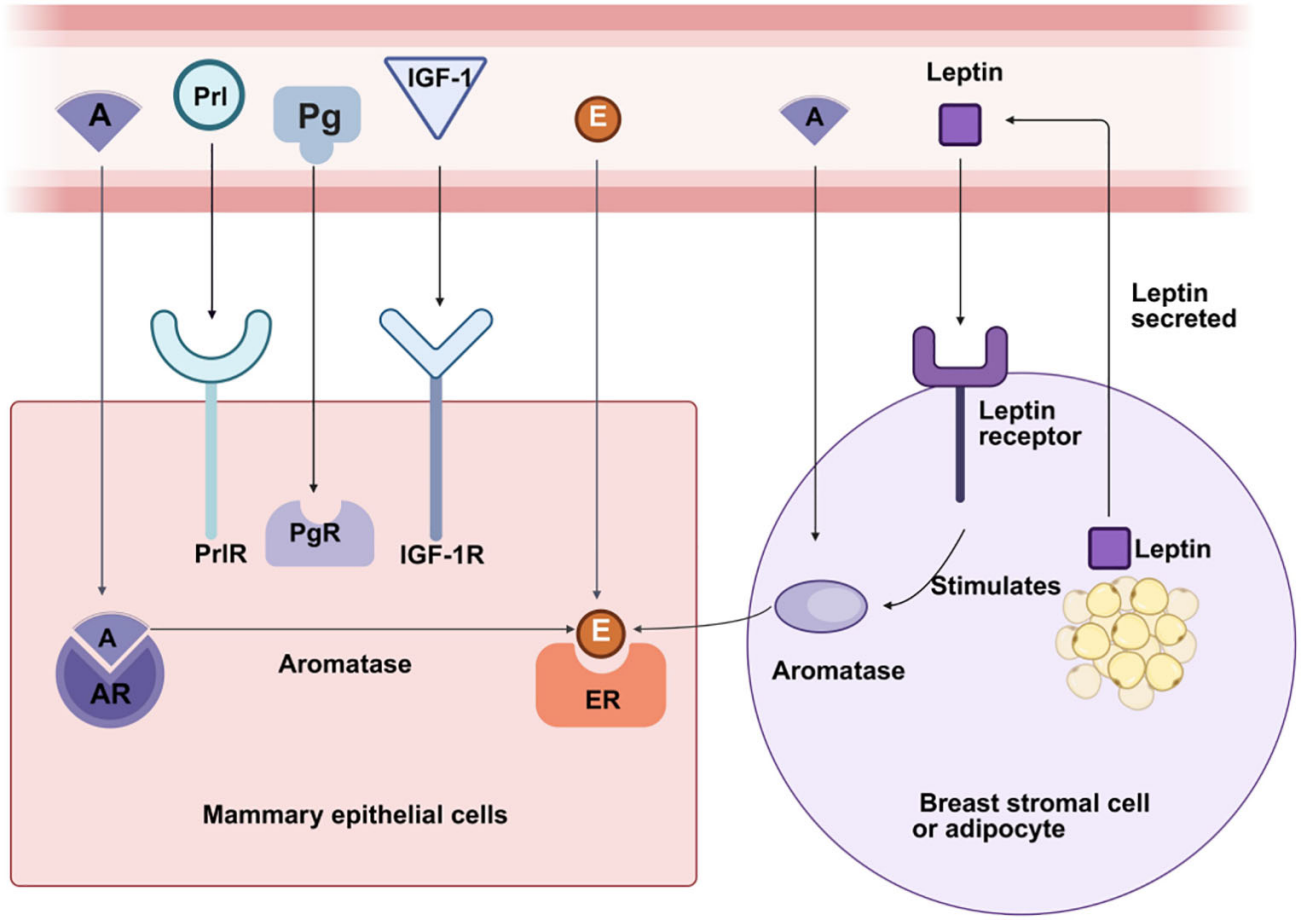
Effects of Different Hormones on the Breast Estrogen, progesterone, prolactim, and other hormones stimulate mammary gland cells, while androgens inhibit mammary gland cells. A, Androgens; AR, Androgen Receptor; Prl, Prolactim; PrlR, Prolactim Receptor; IGF-1, Insulin-like Growth Factor-1; IGF-1R, Insulin-like Growth Factor-1 Receptor; E, Estrogen; ER, Estrogen Receptor.

**Figure 2:**
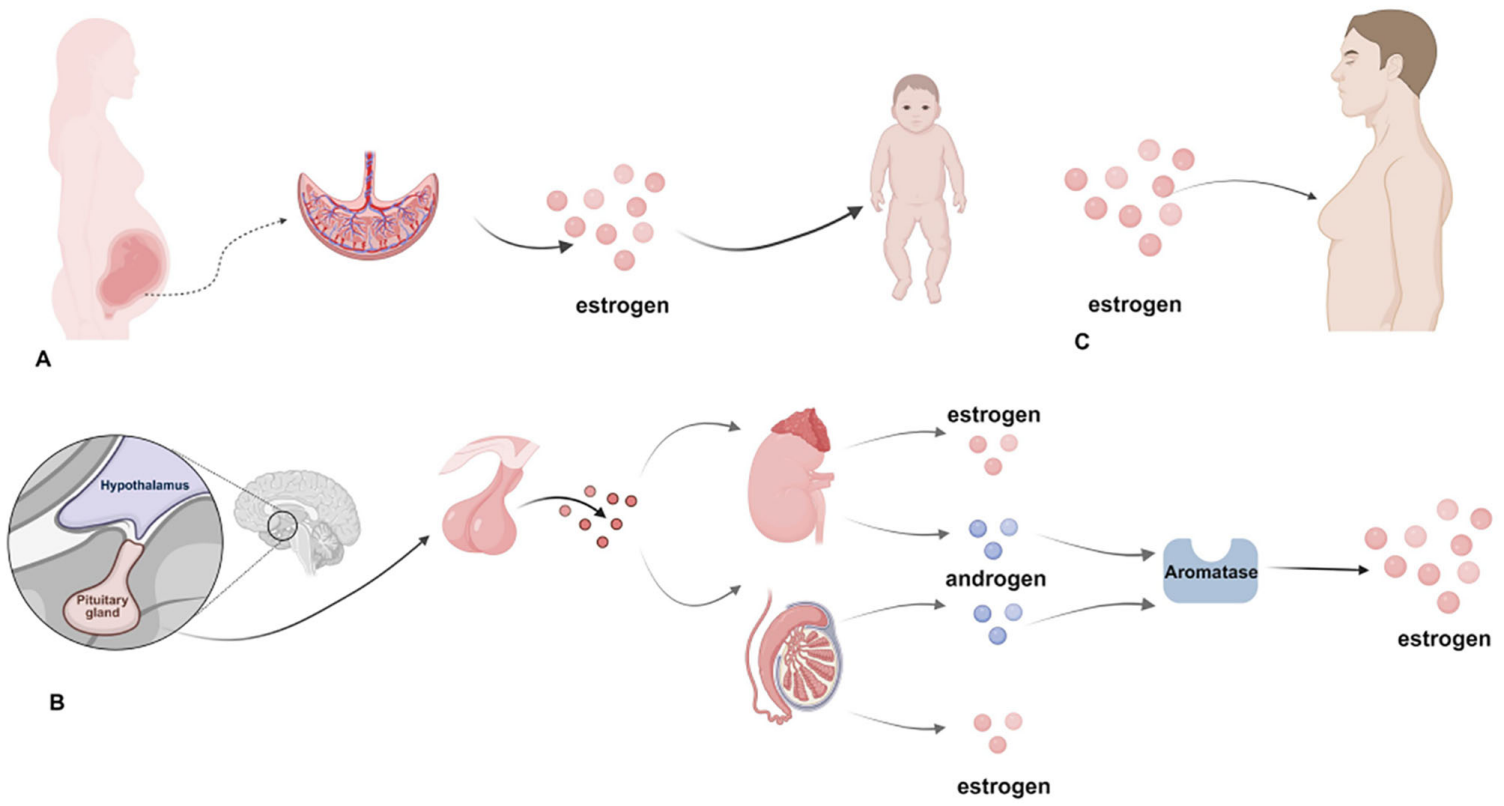
Physiological gynecomastia.

The occurrence of male breast development fundamentally depends on the dynamic balance among multiple hormones, particularly the ratio of estrogen to androgens. Estrogen promotes the proliferation of ductal epithelial cells and stroma in the mammary gland, while androgens (including testosterone and its metabolite dihydrotestosterone) exert an inhibitory effect on mammary tissue (25). When this ratio is imbalanced, gynecomastia may develop even if the absolute levels of either estrogen or androgens remain within normal ranges (16). Beyond estrogen and androgens, other hormones such as insulin-like growth factor-1 (IGF-1), progesterone, and prolactin also play roles in regulating mammary development. IGF-1 promotes the proliferation of mammary epithelial cells, progesterone participates in regulating ductal and stromal development, and prolactin may exacerbate mammary development under certain pathological conditions (16, 26) (see **Figure 1**). Under physiological conditions, mammary development occurs at specific stages: transient enlargement in newborns arises from maternal estrogen crossing the placenta; during puberty, approximately half of adolescent males experience varying degrees of mammary gland development, which typically resolves spontaneously within 1–2 years; in old age, declining androgen secretion coupled with increased aromatase activity in adipose tissue leads to relative estrogen excess, resulting in mammary gland development (27) (see **Figure 2**).

Endocrine-disrupting chemicals (EDCs), ubiquitous in daily life, are core environmental risk factors. Examples include bisphenol A (BPA) and phthalates in plastics and food packaging; atrazine and DDT metabolites in pesticide residues; and pollutants such as polycyclic aromatic hydrocarbons and polychlorinated biphenyls. These pollutants enter the human body via respiration, diet, and skin contact. They disrupt the dynamic balance of estrogen and androgens through mechanisms such as mimicking estrogen, antagonizing androgen receptors, interfering with the activity of hormone synthesis and metabolism enzymes (e.g., aromatase, 5α-reductase), or impairing the function of the hypothalamic-pituitary-gonadal axis (28). Environmentally mediated lifestyle changes also indirectly influence hormonal regulation. For instance, obesity resulting from prolonged exposure to high-fat diets increases estrogen levels by upregulating aromatase expression in adipose tissue. Concurrently, environmentally associated behaviors such as physical inactivity and chronic sleep deprivation may impair hormonal metabolic efficiency. These factors synergize with EDC exposure, collectively increasing the risk of male breast development (29, 30).

Pathological mammary development includes both neoplastic and non-neoplastic conditions. Neoplastic factors, such as tumors of the testes or adrenal glands, may directly secrete estrogen or stimulate its excessive production. Non-neoplastic factors include hypogonadism, hyperthyroidism, liver cirrhosis (which affects estrogen metabolism), and chronic renal failure (leading to impaired hormone clearance) (31). Compared to natural estrogen, BPA exhibits relatively low estrogenic activity (approximately 1/1000). However, BPA concentrations in environmental wastewater, surface water, groundwater, and drinking water far exceed those of natural estrogen. Consequently, BPA’s potential environmental impact warrants serious consideration (32, 33). Existing mammalian toxicology studies indicate that although the doses affecting most mammals are relatively high, the lowest dose producing adverse effects is merely 0.025 μg/kg bw/day. Adverse effects of BPA exposure include weight gain, altered gene expression, carcinogenic effects, and negative impacts on the reproductive system, brain, and nervous system (34). While human toxicological data for BPA remain limited, numerous studies have reviewed epidemiological and human data analyses examining the relationship between BPA exposure and human diseases, including chronic conditions such as diabetes, obesity, reproductive disorders, cardiovascular disease, birth defects, chronic respiratory and renal diseases, and breast cancer (35). BPA may also contribute to human infertility, polycystic ovary syndrome, miscarriage, preterm birth, asthma, and inflammation (18). Furthermore, EDCs from pharmaceutical and environmental exposures can exacerbate these endocrine imbalances by interfering with hormone synthesis, receptor binding, or metabolism, thereby inducing or promoting breast development (36). Notably, the pathogenic doses of EDCs exhibit significant individual variability and cumulative effects, with most requiring only low concentrations to exert endocrine-disrupting actions (37).

## 5 Endocrine disrupting chemicals associated with male breast development

**Figure 3:**
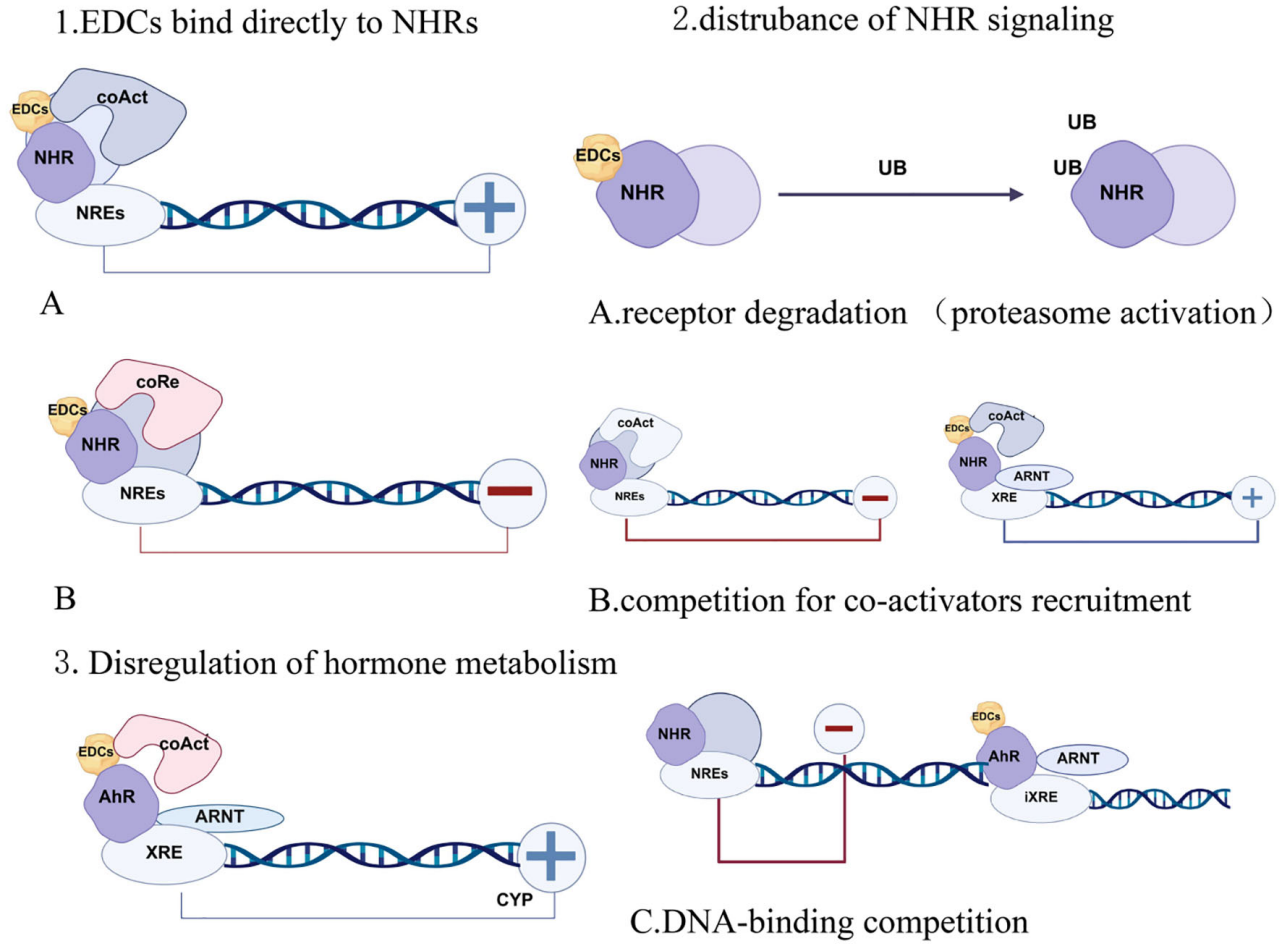
Mechanism of Action of EDCs 1. EDCs can directly blind to NHRs, acting as either **(A)** agonist that induce gene expression or **(B)** antagonist that inhibit receptor activity. 2. EDCs can influence NHR function by inducing **(A)** receptor degradation via proteasome activation, **(B)** competitive recruitment of coactivators, and **(C)** DNA-binding competition. 3. EDCs can disrupt hormone metabolism, primarily by inducing steroid hormone metabolism disorders. **(A)** Activating receptor-mediated proteasome degradation, **(B)** Competing for coactivator recruitment, and **(C)** Competing for DNA binding to influence NHR funxtion. 3. EDCs can disrupt hormone metabolism, primarily by inducing steroid hormone degration. AHR, Aromatic Hormon Receptor; ARNT, Aryl hydrocarbon Receptor Nuclear Translocator; coAct, Coactivator; coRe, Co-repressor; CYP, Cytochrome P450; EDCs, Endocrine Disrupting Chemicals; iXRE, Inhibitory XRE; NHR, Nuclear Hormone Receptor; NRE, NHR Response Element; UB, Ubiquitin; XRE, Xenobiotic Response Element.

Endocrine-disrupting chemicals (EDCs) are typically defined as a class of exogenous chemical substances capable of interfering with the body’s normal hormonal regulatory processes. They may exert effects at multiple stages, including hormone synthesis, secretion, transport, receptor binding, signal transduction, metabolism, and clearance. Through these mechanisms, EDCs can mimic or antagonize endogenous hormone effects, or alter hormonal homeostasis, thereby disrupting developmental, reproductive, and metabolic processes. EDCs may disrupt endogenous hormone function by affecting the activity of 5α-reductase and aromatase (28), leading to imbalances in hormonal equilibrium. Abnormal sex hormone levels can adversely affect sexual development, manifesting as abnormalities in secondary sexual characteristics or gynecomastia (38). Most EDCs can also activate receptors such as the progesterone X receptor (PXR) and estrogen receptor alpha (ERα), and inhibit the androgen receptor (AR) (39). By influencing hormone receptors, they affect hormone action, leading to imbalances in the ratio of sex hormones in the body. Furthermore, EDCs can disrupt the hypothalamic-pituitary-gonadal (HPG) axis through multiple mechanisms, including altering hormone levels and interfering with hormone synthesis and metabolism, thereby causing imbalances in sex hormones (40). In summary, EDCs can disrupt hormonal equilibrium through specific mechanisms. If they affect the absolute or relative concentrations of estrogen and androgens, thereby persistently elevating the estrogen/androgen ratio, this significantly increases the likelihood of inducing gynecomastia in males (see **Figure 3**) (41).

Global production of EDCs has grown exponentially since the 1950s, with the most significant increases observed in fossil fuel-derived EDCs (such as polycyclic aromatic hydrocarbons and phthalates), plastics-related EDCs (such as bisphenol A and PFASs), and pesticide-related EDCs (such as atrazine and DDT). Taking the plastics industry as an example, global annual plastic production surged from approximately 1.5 million tons in 1950 to 460 million tons by 2023 (42, 43). Annual production of EDCs extensively used in plastics manufacturing, such as phthalates and bisphenol A, has now exceeded 10 million tons (44). Among pesticide-derived EDCs, atrazine alone saw global annual usage peak at 33,000 tons (prior to EU prohibition). Even after restrictions, its environmental persistence remains high, with production growth directly increasing human exposure levels to EDCs (45). A study examining bisphenol A exposure levels in the Chinese population from 2004 to 2019 revealed a strong positive correlation between BPA concentrations in human urine and indicators such as waste disposal volumes, domestic sewage treatment, and waste incineration rates (44). Epidemiological monitoring data indicates that BPA metabolites are detectable in over 90% of adult urine samples, while phthalate metabolites have been identified in all urine samples collected globally (46). A study on polycyclic aromatic hydrocarbon (PAH) concentrations in Chinese soil revealed a median increase of 476.8 μg/kg between 2000 and 2020 (47).

Phytoestrogens (PE) are a class of non-steroidal compounds derived from plants (such as soybeans, flaxseed, and nuts). Due to the presence of a phenolic hydroxyl group in their molecular structure (similar to 17β-estradiol), they can bind to estrogen receptors (ERα, ERβ) and G protein-coupled estrogen receptors (GPER), exerting estrogenic or anti-estrogenic effects. This aligns with the core definition of EDCs as “substances that disrupt hormone action”. However, their effects are bidirectional and dose-dependent. At typical dietary intake levels, they often exhibit potential protective effects. Compared to synthetic EDCs, they offer advantages such as controllable exposure, rapid metabolism, and lower effect potency (48).

With advances in science and technology and societal development, EDCs are now ubiquitous in everyday products. Humans may be exposed to EDCs from the embryonic stage via multiple routes, including the skin, respiratory tract, and digestive tract (6). Vertical exposure (i.e., maternal-fetal placental transfer) constitutes the primary pathway for embryonic EDC exposure, with hazards that are long-term and irreversible: EDCs ingested by the mother during pregnancy via diet, respiration, or skin contact (such as bisphenol A, phthalates, polychlorinated biphenyls, etc.) can cross the placental barrier into the fetus, disrupting the normal development of the embryonic gonads and endocrine system (49). This not only causes hormonal imbalances in the fetus, increasing the risk of postnatal conditions such as gynecomastia, cryptorchidism, and abnormal sperm quality but also may alter gene expression through epigenetic modifications (such as DNA methylation and histone modifications), enabling transgenerational transmission (50). For instance, male offspring of pregnant mice exposed to bisphenol A exhibited abnormal proliferation of mammary epithelial cells, leading to enlarged breasts and a significantly increased risk of mammary carcinoma in adulthood (18). Furthermore, placental exposure to EDCs may impair the developmental maturation of the fetal hypothalamic-pituitary-gonadal axis, disrupting hormonal regulation during puberty. This amplifies the sensitivity of breast tissue to hormones, laying the groundwork for abnormal breast hyperplasia or carcinogenesis in adulthood. Although excessive exposure to EDCs is known to contribute to various diseases, such as reproductive abnormalities, metabolic disorders, endocrine dysfunction, and cancers including breast and prostate cancer (5), EDCs also exhibit a “cocktail effect”, wherein two distinct EDCs or an EDC combined with an endogenous hormone may simultaneously bind to receptors and induce positive synergistic effects. This means that the adverse reactions of EDC mixtures exceed the sum of the negative impacts produced by each EDC individually (51). However, current research offers limited evidence linking EDCs specifically to male breast development.

This paper examines the impact of EDCs on male breast development, focusing on certain EDCs previously reported in association with this condition and several EDCs commonly encountered in daily life. It aims to elucidate the mechanisms by which EDCs influence male breast development, thereby providing a reference for further research into these specific effects. This understanding will assist in formulating novel intervention and prevention strategies to reduce the incidence of male breast development.

### 5.1 Bisphenol compounds

Bisphenol compounds, such as bisphenol A (BPA), bisphenol S (BPS), bisphenol C (BPC), bisphenol F (BPF), bisphenol AF (BPAF), tetrabromobisphenol, nonylphenol, and octylphenol, are plasticizers used globally in the manufacture of everyday items (52). Experimental studies indicate that bisphenols can alter hormone levels by binding to specific receptors such as the estrogen receptor (ER) and androgen receptor (AR). They may also influence endogenous estrogen and androgen concentrations by affecting the activity of enzymes involved in sex hormone synthesis or metabolism (53). Furthermore, bisphenols target mRNA gene expression, disrupting the HPG axis via KiSS1 and ERα, thereby influencing the body’s gonadotropin content (54). Through these pathways, bisphenols affect estrogen/androgen levels to induce gynecomastia. Regarding inflammatory responses, evidence suggests that bisphenol exposure is associated with alterations in the inflammatory microenvironment (55). This article uses bisphenol A and nonylphenol as representative examples of bisphenol compounds.

#### 5.1.1 bisphenol A

Bisphenol A (BPA) is a high-production-volume chemical globally and one of the most extensively studied endocrine-disrupting chemicals (EDCs). Initially synthesized as a synthetic estrogen (xenoestrogen), it is now a key monomer in producing polycarbonate plastics and epoxy resins. It is commonly found in everyday items including optical products, electronic devices, medical equipment, and reusable plastic utensils. BPA has been detected in various environmental media, including drinking water, air, and dust (56). ely low concentrations (57, 58). It may also exert agonist effects on G protein-coupled receptors (GPCRs) and γ-estrogen-related receptors (ERRc) (56, 59–61), subsequently contributing to disorders such as menstrual irregularities and ovarian dysfunction in females, as well as diminished sperm quality in males (62–64). Although studies have not explicitly documented BPA’s direct impact on male mammary development, it may increase susceptibility by altering estrogen levels. Furthermore, experiments observed that mammary epithelial cells in male CD-1 mice exposed to BPA during early life retained long-term responsiveness (18).

Given BPA’s potential health hazards, the European Union banned its use in infant feeding bottles in 2011. Substitutes such as bisphenol S (BPS) and bisphenol F (BPF) subsequently emerged. However, due to structural similarity, these alternatives also exhibit hormonal activity, potentially exerting estrogenic, anti-estrogenic, androgenic, and anti-androgenic effects (65). Further research is required to assess their risks to human health. Bisphenol S (BPS) and bisphenol F (BPF), as common substitutes for BPA, both exhibit clear endocrine-disrupting activity. BPS primarily disrupts thyroid function, affects neural and embryonic development, and interferes with energy metabolism. BPF, meanwhile, predominantly impacts reproductive organ development, interferes with androgen receptors, and may induce obesity. Both compounds also exhibit immunotoxicity, and mixed exposure may produce additive or even synergistic effects, posing potential health threats to sensitive populations (66).

Within the body, BPA is primarily glucuronidated via hepatic microsomes through catalysis by uridine diphosphate-glucuronosyltransferase (UGT) isoforms, subsequently being rapidly excreted via feces and urine. Consequently, BPA exposure levels are typically determined by analyzing urine samples (67). Results from *in vitro* and *in vivo* studies indicate that BPA exhibits potent efficacy in non-genomic activation of adipogenic transcription factors (68), upregulation of adipogenic genes (69), and lipid accumulation (70). Within human adipose tissue, BPA stimulates the release of pro-adipogenic cytokines, and obesity itself is a contributing factor to male breast development. In obese individuals, increased aromatase expression and activity may both be implicated in the development of gynecomastia. Furthermore, BPA may also contribute to alterations in glucose homeostasis (71); animal models indicate that BPA can increase insulin secretion (72). Abnormal insulin levels or insulin resistance can lead to obesity, diabetes, and metabolic syndrome. Additionally, insulin resistance and hyperinsulinemia may drive gynecomastia by increasing aromatase activity, altering sex hormone-binding globulin (SHBG) levels, and elevating the estradiol/testosterone (E2/T) ratio (73). BPA also induces inflammatory responses via the MAPK family and associated transcription factors (e.g., AP-1, NF-κB, and STAT family), while further influencing immune responses by affecting various immune cells (e.g., CD4+ T cells, B cells, macrophages, etc.) (74). Bisphenol A may thus exacerbate male breast development by provoking inflammatory reactions.

#### 5.1.2 Nonyl phenol

Nonylphenol belongs to the alkylphenol ethoxylates (APEs) within the non-ionic surfactant family and is used in textile printing and dyeing auxiliaries, lubricant additives, pesticide emulsifiers, resin modifiers, and stabilizers. It is a xenoestrogen formed during the degradation of ethoxylated nonylphenol (NPEO) (75), exhibiting persistence, lipophilicity, and greater bioaccumulation potential than NPEO (76). NP shares structural similarity with the natural hormone 17β-estradiol, enabling it to compete for receptor binding sites and interact with the estrogen receptors ERα and ERβ. However, not all nonylphenol isomers induce estrogenic activity; the estrogenic effect of NP varies by isomer. Among these, 4-(1′,1′-dimethyl-2′-ethylpentyl)-phenol (NP7) exhibits potent estrogenic activity. Conversely, other studies indicate that while NP demonstrates estrogenic activity, it also possesses anti-estrogenic effects (77), potentially influenced by concentration, exposure duration, and interactions with other hormonal levels in the organism.

Furthermore, NP exhibits anti-androgenic effects. Through multi-step activation of androgen receptors, it may disrupt normal male development and the essential functions of androgens in the reproductive system. This can lead to reduced circulating testosterone levels, diminished antioxidant enzyme activity in sperm, testicular structural disruption, and increased apoptosis in supporting cells (78). In males, where androgens predominate, these hormones determine fundamental physiological characteristics. Androgens (primarily testosterone and dihydrotestosterone) inhibit estrogen-induced mammary proliferation through multiple signaling pathways centered on the androgen receptor (AR). These include direct transcriptional regulation of proliferation-related genes, antagonism of estrogen receptor signaling, and inhibition of the PI3K/Akt/mTOR and MAPK pathways, while simultaneously promoting estrogen inactivation and abnormal apoptosis. These pathways synergistically maintain the normal state of the male mammary gland, and impairment of their function may trigger mammary development. Androgens can inhibit mammary proliferation under estrogen exposure through a series of signaling pathways, hence male breasts typically do not undergo further development (79).

Studies have demonstrated that exposure to nonylphenol in male mice reduces serum testosterone levels, with key genes involved in steroidogenesis and their corresponding proteins also being downregulated (80). These investigations indicate that nonylphenol may influence estrogen or androgen levels in the body, though the precise regulatory mechanisms remain incompletely understood. Although no studies have yet reported on the effects of nonylphenol on male breast development, if its influence on estrogen and androgen levels leads to an elevated estrogen/androgen ratio, it may potentially induce male breast development and alterations in reproductive function.

### 5.2 Phthalates

Phthalates (PAEs) are widely used in personal care products and as plasticizers in plastics. Among PAEs, di(2-ethylhexyl) phthalate (DEHP) and its primary metabolite mono(2-ethylhexyl) phthalate (MEHP) are commonly used as plasticizers in polyvinyl chloride (PVC) and other plastic products. They enhance plastic elasticity and toughness, finding extensive application in the plastics industry. Exposure to DEHP may induce male reproductive toxicity, developmental toxicity, and hepatotoxicity (81). Phthalates are classified as EDCs due to their interference with normal hormonal homeostasis (82). Research indicates that phthalates exhibit anti-estrogenic, anti-androgenic, anti-progesterone, and anti-thyroid activities (83). The general population primarily encounters phthalates through ingestion of contaminated food and inhalation via the respiratory tract. Following uptake by intestinal epithelial cells, phthalates undergo hydrolysis into monoalkyl phthalate esters. Furthermore, extensive and continuous hydrolysis and oxidation of phthalates in the body lead to the formation of numerous secondary metabolites (84). Phthalates undergo rapid metabolism and are almost entirely eliminated via urine. Measuring exposure levels is crucial for subsequent studies investigating phthalate impacts on human health, and numerous research reports indicate that urinary metabolite concentrations serve as the most reliable indicator for assessing phthalate exposure.

However, past research on phthalates has primarily focused on individual phthalate esters, whereas real-world exposure to EDCs typically involves complex mixtures of these compounds. A study examining phthalate exposure and endometrial hyperplasia in mice demonstrated that prolonged exposure to phthalate mixtures enhances estrogen signaling, inflammation, and epithelial cell proliferation. Following phthalate exposure, levels of the estrogen-regulated genes Muc1, Fgf9, Ccn1, Cx43, and Hif2α increased (85).

Excessive estrogen stimulation exhibits carcinogenic potential in human breast tissue. Research by Dairkee SH et al. demonstrated that intervention against phthalate exposure significantly reversed cancer-associated phenotypes in mammary tissue, concurrently detecting a marked reduction in urinary phthalate metabolites (86). Furthermore, epidemiological studies indicate an association between phthalate exposure and adverse health outcomes in humans, particularly concerning development and reproduction. These endocrine-disrupting effects appear linked not only to anti-androgenic activity (87) but also to estrogenic and anti-estrogenic activities primarily mediated by phthalates. Combined exposure to phthalates and their metabolites leads to elevated estradiol levels and reduced total testosterone (TT) levels. Furthermore, estradiol concentrations correlate positively with obesity, whereas total testosterone (TT) levels correlate negatively with obesity (88).

Although no studies currently exist on the effects and mechanisms of phthalates in male breast development, existing research evidence indicates that male mammary gland development is primarily associated with an imbalance in the estrogen/androgen ratio, and that obesity is correlated with male breast development (36, 89, 90). Therefore, there is reason to consider phthalate esters (PAEs) as potential risk factors for male breast development, with exposure potentially inducing such changes. Relevant studies in females provide indirect support for this mechanism: multiple epidemiological and experimental studies in females confirm that phthalate exposure increases the estradiol/testosterone ratio by inhibiting androgen synthesis and enhancing aromatase activity, while simultaneously promoting adipose tissue accumulation (exacerbating obesity-related mammary hyperplasia). This consequently elevates the risk of mammary hyperplasia and breast cancer (86, 88); the core mechanisms involve antagonizing androgen receptors, activating estrogen signaling pathways, and disrupting lipid metabolism. These closely align with key regulatory mechanisms in male breast development (hormonal imbalance and obesity-mediated proliferative effects), suggesting that phthalates’ hormonal interference in breast tissue exhibits gender universality. This indirectly corroborates their potential to induce male breast development via similar pathways.

### 5.3 Polycyclic aromatic hydrocarbons

Polycyclic aromatic hydrocarbons (PAHs) are ubiquitous pollutants, posing a threat to ecosystems and human health as organic contaminants. Their physical and chemical properties enable accumulation within living organisms and confer high mobility in the environment. Sources of PAH exposure in daily life are extensive, including inhalation, dietary intake, and skin contact, with dietary consumption representing a significant route of non-occupational exposure (91). The most prevalent components of PAHs are found in petrol and vehicle exhaust fumes, making exposure to these substances difficult to avoid. Certain PAH metabolites act as EDCs by exerting estrogenic and anti-estrogenic activities, particularly hydroxylated PAHs, which share a structural conformation with 17β-estradiol (92). Female Wistar rats exposed to four distinct PAH concentrations (benzo[a]anthracene [BaP], fluoranthene [Fla], and benzo[k]fluoranthene [BkF]) for 22 days exhibited enhanced weak expression of the α-estrogen receptor and a significant increase in testosterone 6β-hydroxylase activity, indicating the potential of PAHs to induce estrogenic activity *in vivo* (93).

Benzo[a]pyrene (B[a]P) is one of the most representative polycyclic aromatic hydrocarbons; this section uses B[a]P as an example to introduce PAHs. B[a]P is a common polycyclic aromatic hydrocarbon in daily life, present in cigarette smoke, vehicle exhaust, and foodstuffs, particularly smoked and barbecued foods. The primary routes of B[a]P exposure are contaminated food and air (94). B[a]P is recognized as a reproductive and developmental toxin, with extensive research confirming its carcinogenic properties; exposure to B[a]P can lead to cancer development. PAH exposure alters the epigenetic and transcriptional regulation of genes within the estrogen receptor α (Erα) pathway, thereby contributing to the initiation and progression of mammary tumors (95, 96). Furthermore, modifications in the epigenetic and transcriptional regulation of certain Erα pathway genes observed in pregnant female mice may drive the changes subsequently noted in their offspring and grandprogeny (97). Although fundamental differences exist between breast cancer and male breast development (98), the activation of the Erα pathway by B[a]P induces hormonal alterations in both conditions. Consequently, an inherent link must exist between male breast development and breast cancer. If B[a]P can induce breast cancer, its association with male breast development warrants scrutiny.

### 5.4 polychlorinated biphenyls

Polychlorinated biphenyls (PCBs) are toxic environmental pollutants, a class of man-made organic chemicals whose biphenyl structure may be substituted by 1 to 10 chlorine atoms. Due to multiple substitution positions, PCBs comprise 209 distinct congeners (45). PCBs possess excellent dielectric properties, are stable against chemical and thermal degradation, are unaffected by light, and are non-flammable (99). These characteristics led to their widespread use across various industrial and commercial sectors, most notably as insulating fluids in transformers and capacitors, and as additives in the manufacture of paints, plastics, and carbonless copy paper (100). Consumption of contaminated food is generally recognized as the primary source of human exposure to PCBs. In 2015, the International Agency for Research on Cancer classified PCBs as a Group 1 carcinogen (carcinogenic to humans), further highlighting their hazards to human health as EDCs. Despite bans on PCB production and use, small quantities persist due to incomplete combustion during waste incineration or industrial processes. Human exposure to PCBs in the atmosphere, soil, and aquatic environments remains a significant health risk.

Lerro and Jones described a potential association between PCB exposure and thyroid cancer (101). PCBs have also been demonstrated to be weakly estrogenic organic compounds associated with testicular cancer (102), prostate cancer (103), and breast cancer. Furthermore, exposure to PCBs may increase the aggressiveness and metastasis of breast cancer in women, thereby worsening their prognosis (104). PCBs exhibit both estrogenic activity and anti-androgenic properties (105), and research into the pathogenic effects of PCBs has primarily focused on their activity at estrogen receptors. Among the 209 PCBs, PCB-129 exhibits the strongest binding affinity to the estrogen receptor. In certain circumstances, PCBs may bind to the receptor more effectively than natural chemicals, potentially disrupting or blocking normal biochemical processes (106). However, most PCB-estrogen research has centered on females. If PCBs influence estrogen receptor activity in males, thereby altering the body’s estrogen/androgen ratio, they could potentially induce male breast development. Further investigation into the association between PCBs and male breast development is warranted.

### 5.5 Pesticides

Despite ongoing societal progress and increasingly modernized lifestyles, humanity continues to rely extensively on pesticides such as herbicides and insecticides in agricultural production. These substances pose significant hazards to human health, with some acting as EDCs that can alter the body’s endocrine system function, thereby causing adverse health effects in individuals or their offspring. Humans primarily encounter pesticides through ingestion, inhalation, or dermal absorption, with pesticide residues in food constituting the principal exposure source. Urine, blood/serum, and hair are used as the primary biomonitoring matrices (107). This section selects several representative pesticides with EDC characteristics to investigate their respective associations with gynecomastia.

#### 5.5.1 Herbicide atrazine

Atrazine (ATZ) is an agricultural herbicide that enhances crop yields and maintains crop health (108). Following gastrointestinal absorption, atrazine is primarily metabolized in the liver by cytochrome P450 (CYP450) enzymes (109). ATZ and its major metabolites are eliminated through conjugation with glutathione (GSH) and excreted via urine and feces (110, 111). ATZ exposure has been associated with reproductive system dysfunction in both animals and humans. The reproductive system, as part of the endocrine system regulated by the hypothalamic-pituitary-gonadal (HPG) axis, is affected by atrazine through this axis. Atrazine influences androgens such as testosterone and disrupts the endocrine system (112). *In vivo* studies demonstrate that male rat exposure to atrazine induces inflammatory and oxidative stress responses (113). Furthermore, research indicates that chronic environmental exposure to atrazine alters the expression of steroidogenesis genes in mouse testes (114), subsequently reducing testosterone production in juvenile rats. ATZ directly inhibits expression of the Srd5α1 gene encoding type 1 5α-reductase (115), thereby qualifying it as an anti-androgenic EDC. By suppressing 5α-reductase activity, ATZ reduces the conversion of testosterone to 5α-dihydrotestosterone (DHT), consequently lowering DHT levels. ATZ also exhibits estrogenic effects by increasing expression of the Cyp19a1 gene encoding aromatase. Atrazine promotes aromatase activity, facilitating the conversion of testosterone to estrogen and consequently elevating estrogen levels (115). Given ATZ’s potential to lower testosterone levels and enhance aromatase activity, it may induce male breast development.

The effects of atrazine on the male reproductive system have been extensively documented (113, 114). Due to its potential endocrine-disrupting effects (such as inducing reproductive dysfunction), the European Union banned the use of atrazine in 2004. Despite the EU’s early regulatory measures, due to the compound’s long half-life, atrazine continues to cause significant contamination of groundwater and surface water in countries and regions such as the United States (116). Consequently, further restrictions on the use of such pesticides and human exposure are warranted.

#### 5.5.2 Pesticide DDT and its analogues

Insecticides are chemical substances of natural or synthetic origin used to eliminate pests and insects, being ubiquitous in daily life: organochlorine pesticides such as dichlorodiphenyltrichloroethane (DDT), organophosphorus pesticides such as dichlorvos (DDVP), pyrethroid insecticides including cypermethrin, and neonicotinoids like thiamethoxam and imidacloprid. They are indispensable in agricultural production due to their role in enhancing crop yields and protecting crops from diseases and pests (117). However, their high toxicity, environmental persistence, high bioaccumulation potential, and low biodegradability continue to pose significant environmental hazards.

Taking the organochlorine pesticide DDT as an example, this section illustrates the link between EDCs and male breast development. Due to its severe ecological damage, DDT has been comprehensively banned in most nations, though it remains used as an insecticide for malaria control in certain developing countries (118). DDT metabolites bind to lipids and accumulate in adipose tissue (119). Their persistent presence in the human body can affect the reproductive system by altering sex hormone levels, leading to stillbirths, birth defects, spontaneous abortions, and infertility. DDT exhibits anti-androgenic and estrogen-like properties. A rat study indicated that daily exposure during fetal and postnatal developmental stages may alter both male and female sex hormone levels. This effect is likely mediated by DDT’s direct interference and the involvement of the hypothalamic-pituitary axis (120). Another study indicated that DDT’s endocrine disruption mechanism involves not only competing with testosterone for androgen-binding receptors, thereby impairing receptor signaling in target cells, but may also occur through promoting estrogen synthesis pathways (121). These alterations in sex hormone production and secretion suggest that early-life exposure to DDT may predispose individuals to reproductive and systemic disorders later in life. Excessive estrogen secretion and disrupted testosterone/estradiol ratios increase the risk of feminization (e.g., gynecomastia) and may also induce metabolic abnormalities, estrogen-dependent tumors, and cardiovascular diseases.

### 5.6 Essential oils such as lavender oil and tea tree oil

It is widely believed that essential oils are derived from nature and safer than chemical drugs, yet this perception has limitations. Certain essential oils—lavender oil and tea tree oil being prime examples—have been demonstrated to potentially act as EDCs, influencing male breast development (38). The associated mechanisms and controversies warrant thorough investigation.

From a compositional perspective, lavender oil and tea tree oil have complex chemical structures. Their core constituents include terpenoid hydrocarbons (monoterpenes, sesquiterpenes, etc.) and oxygenated compounds (linalool, linalyl acetate, etc.). This multi-component mixture is the fundamental basis for their physiological activity and potential risks (38). Clinical cases provide direct evidence for their endocrine-disrupting effects: reports indicate that three prepubescent boys with male breast development had all used over-the-counter personal care products (such as skincare items and fragrances) containing lavender or tea tree oil repeatedly and topically for extended periods (122). With other identifiable causative factors ruled out, this suggests an association between such essential oils and abnormal breast development. Further mechanistic studies indicate that extracts of lavender oil and tea tree oil can influence the activity of CYP17A1 (a key enzyme in steroid hormone synthesis) and aromatase CYP19A1 (18). CYP17A1 participates in androgen synthesis, while CYP19A1 is responsible for converting testosterone into estrogen. Disruption of either function directly disrupts the body’s estrogen-androgen balance, leading to a relative increase in estrogen levels (38). This, in turn, creates conditions conducive to mammary proliferation, aligning closely with the core pathogenic mechanism of male breast development.

However, the endocrine-disrupting effects of essential oils remain controversial. Recent studies have found that the individual components linalool (Lin) and linalyl acetate (LinAc) present in essential oils did not exhibit endocrine-disrupting activity in either *in vitro* or *in vivo* experiments. Furthermore, no evidence has been established linking them to precocious puberty in children or prepubertal gynecomastia in males (123). This conclusion does not negate the potential risks associated with essential oils as a whole. The core reason lies in the fact that linalool and linalyl acetate are merely individual components within the complex mixtures of essential oils. The biological effects of essential oils often stem from the synergistic interactions of multiple components; the absence of activity in a single component does not imply the mixture is devoid of effects. Current research gaps remain significant: it remains unclear precisely which component(s) (rather than individual constituents) within essential oil mixtures mediate endocrine disruption. Systematic data supporting specific target mechanisms, dose-response relationships, efficiency of transdermal absorption during topical application, and metabolic pathways in the body are notably lacking.

In summary, existing evidence suggests that essential oils such as lavender oil and tea tree oil may potentially contribute to male breast development by interfering with hormone synthase activity and disrupting estrogen-androgen balance. However, the underlying mechanisms require further validation. Future research should focus on the holistic effects of complex essential oil mixtures rather than the isolated actions of individual components. This includes identifying key active constituents responsible for endocrine disruption and elucidating their specific molecular mechanisms. Concurrently, risk assessments should be optimized by incorporating clinical exposure scenarios to provide scientific grounds for the safe use of essential oils and the prevention of associated health risks.

## 6 Conclusion

Endocrine-disrupting chemicals (EDCs) are a class of chemicals widely present in the environment that can mimic or interfere with the body’s endocrine system, thereby affecting normal physiological functions. In this review, we explore in detail the effects of EDCs on male breast development. Existing research indicates that certain EDCs may act as potential risk factors for male breast development. By mimicking estrogenic or anti-androgenic effects, they participate in regulating mammary development, potentially leading to abnormal breast growth in males and even the onset of breast cancer.

Given the ubiquitous presence of EDCs in the environment, widespread human exposure is a fundamental reality, with virtually no truly “zero-exposure” populations. This poses significant challenges for related research. Specifically, it is difficult to clearly elucidate the chemical interactions between EDCs and biomolecules, as well as their causal relationships in disease onset and progression. Current research strategies primarily employ single-EDC exposure models, failing to adequately reflect the combined effects of mixed exposure to multiple EDCs encountered in reality. Different classes of EDCs may interact additively or synergistically, complicating both the prediction of health effects and the establishment of causal evidence linking specific EDCs to endocrine disruption. Furthermore, effective exposure monitoring methods for the vast array of known or suspected EDCs remain lacking, as do detection techniques capable of accurately capturing mixture effects.

Despite progress in this field, research on the relationship between EDCs and male breast development remains relatively limited, with specific mechanisms of action yet to be fully elucidated. Future studies should focus on unraveling the underlying biological mechanisms and actively exploring strategies to prevent the adverse effects of EDCs on male breast development.

## References

[1] Prasad P Bennett A Speirs V Shaaban AM . Morphological features and immunohistochemical profiling of male breast gynaecomastia; A large tissue microarray study. Front Oncol. (2022) 12:875839. doi: 10.3389/fonc.2022.875839, PMID: 35814372 PMC9261459

[2] Reinehr T Kulle A Barth A Ackermann J Lass N Holterhus PM . Sex hormone profile in pubertal boys with gynecomastia and pseudogynecomastia. J Clin Endocrinol Metab. (2020) 105. doi: 10.1210/clinem/dgaa044, PMID: 31996898

[3] Marcus R Korenman SG . Estrogens and the human male. Annu Rev Med. (1976) 27:357–70. doi: 10.1146/annurev.me.27.020176.002041, PMID: 779604

[4] Braunstein GD . Aromatase and gynecomastia. Endocr Relat Cancer. (1999) 6:315–24. doi: 10.1677/erc.0.0060315, PMID: 10731125

[5] Trasande L Sargis RM . Endocrine-disrupting chemicals: Mainstream recognition of health effects and implications for the practicing internist. J Internal Med. (2023) 295:259–74. doi: 10.1111/joim.13748, PMID: 38037246 PMC11457725

[6] Pan J Liu P Yu X Zhang Z Liu J . The adverse role of endocrine disrupting chemicals in the reproductive system. Front Endocrinol. (2024) 14. doi: 10.3389/fendo.2023.1324993, PMID: 38303976 PMC10832042

[7] Vandenberg LN . Endocrine disrupting chemicals and the mammary gland. Adv Pharmacol. (2021) 92:237–77. doi: 10.1016/bs.apha.2021.04.005, PMID: 34452688

[8] Lee SW Ahn SH Myung Y . Secondary Genioplasties for the Treatment of Chin Deformities After Orthognathic Surgery in Asian Women: Defining the Aesthetic Importance of Managing the Chin Shape in Orthognathic Surgery. Ann Plast Surg. (2025) 95:744–51. doi: 10.1097/SAP.0000000000004482, PMID: 25710556

[9] Ordaz DL Thompson JK . Gynecomastia and psychological functioning: A review of the literature. Body Image. (2015) 15:141–8. doi: 10.1016/j.bodyim.2015.08.004, PMID: 26408934

[10] Mercan Isik C Ozturk M Bestas A . Gynecomastia and adolescence: Psychological effects of social appearance anxiety and peer bullying. J Pediatr Nurs. (2025) 83:23–9. doi: 10.1016/j.pedn.2025.04.017, PMID: 40279823

[11] Ford HC Cooke RR Keightley EA Feek CM . Serum levels of free and bound testosterone in hyperthyroidism. Clin Endocrinol (Oxf). (1992) 36:187–92. doi: 10.1111/j.1365-2265.1992.tb00956.x, PMID: 1568351

[12] Narula HS Carlson HE . Gynecomastia. Endocrinol Metab Clin North Am. (2007) 36:497–519. doi: 10.1016/j.ecl.2007.03.013, PMID: 17543732

[13] Braunstein GD . Gynecomastia. N Engl J Med. (1993) 328:490–5. doi: 10.1056/NEJM199302183280708, PMID: 8421478

[14] Magro G Gangemi P Villari L Greco P . Deciduoid-like stromal cells in a diabetic patient with bilateral gynecomastia: a potential diagnostic pitfall. Virchows Arch. (2004) 445:659–60. doi: 10.1007/s00428-004-1098-x, PMID: 15378358

[15] Fox S Speirs V Shaaban AM . Male breast cancer: an update. Virchows Arch. (2022) 480:85–93. doi: 10.1007/s00428-021-03190-7, PMID: 34458944

[16] Johnson RE Murad MH . Gynecomastia: pathophysiology, evaluation, and management. Mayo Clin Proc. (2009) 84:1010–5. doi: 10.1016/S0025-6196(11)60671-X, PMID: 19880691 PMC2770912

[17] Ismail AA Barth JH . Endocrinology of gynaecomastia. Ann Clin Biochem. (2001) 38:596–607. doi: 10.1258/0004563011900993, PMID: 11732643

[18] Vandenberg LN Schaeberle CM Rubin BS Sonnenschein C Soto AM . The male mammary gland: a target for the xenoestrogen bisphenol A. Reprod Toxicol. (2013) 37:15–23. doi: 10.1016/j.reprotox.2013.01.002, PMID: 23348055 PMC3998714

[19] Ahn C Jeung E-B . Endocrine-disrupting chemicals and disease endpoints. Int J Mol Sci. (2023) 24. doi: 10.3390/ijms24065342, PMID: 36982431 PMC10049097

[20] Calafat AM Ye X Wong LY Reidy JA Needham LL . Exposure of the U.S. population to bisphenol A and 4-tertiary-octylphenol: 2003–2004. Environ Health Perspect. (2008) 116:39–44. doi: 10.1289/ehp.10753, PMID: 18197297 PMC2199288

[21] Kumar M Sarma DK Shubham S Kumawat M Verma V Prakash A . Environmental endocrine-disrupting chemical exposure: role in non-communicable diseases. Front Public Health. (2020) 8:553850. doi: 10.3389/fpubh.2020.553850, PMID: 33072697 PMC7541969

[22] Acharya SV . Clinical features, presentation and hormonal parameters in patients with pubertal gynecomastia. J Family Med Prim Care. (2021) 10:648–51. doi: 10.4103/jfmpc.jfmpc_1987_20, PMID: 34041055 PMC8138374

[23] Dickson G . Gynecomastia. Am Fam Physician. (2012) 85:716–22., PMID: 22534349

[24] Bannayan GA Hajdu SI . Gynecomastia: clinicopathologic study of 351 cases. Am J Clin Pathol. (1972) 57:431–7. doi: 10.1093/ajcp/57.4.431, PMID: 5012934

[25] Colamaio EB Russo L Bianco AF Califano D Chiappetta PP Troncone G . Translational highlights from the journal of clinical endocrinology & metabolism. Mol Endocrinol. (2011) 25:1978–9. doi: 10.1210/mend.25.11.zmg1978, PMID: 22034475 PMC5417180

[26] Macias H Hinck L . Mammary gland development. WIREs Dev Biol. (2012) 1:533–57. doi: 10.1002/wdev.35, PMID: 22844349 PMC3404495

[27] Mcnally S Stein T . Overview of mammary gland development: A comparison of mouse and human. Methods Mol Biol. (2017) 1501:1–17. doi: 10.1007/978-1-4939-6475-8_1, PMID: 27796946

[28] Yilmaz B Terekeci H Sandal S Kelestimur F . Endocrine disrupting chemicals: exposure, effects on human health, mechanism of action, models for testing and strategies for prevention. Rev Endocrine Metab Disord. (2019) 21:127–47. doi: 10.1007/s11154-019-09521-z, PMID: 31792807

[29] Williams G . Aromatase up-regulation, insulin and raised intracellular oestrogens in men, induce adiposity, metabolic syndrome and prostate disease, via aberrant ER-alpha and GPER signalling. Mol Cell Endocrinol. (2012) 351:269–78. doi: 10.1016/j.mce.2011.12.017, PMID: 22233684

[30] Newbold RR Padilla-Banks E Jefferson WN . Environmental estrogens and obesity. Mol Cell Endocrinol. (2009) 304:84–9. doi: 10.1016/j.mce.2009.02.024, PMID: 19433252 PMC2682588

[31] Narula HS Carlson HE . Gynaecomastia–pathophysiology, diagnosis and treatment. Nat Rev Endocrinol. (2014) 10:684–98. doi: 10.1038/nrendo.2014.139, PMID: 25112235

[32] Liu ZH Kanjo Y Mizutani S . Removal mechanisms for endocrine disrupting compounds (EDCs) in wastewater treatment - physical means, biodegradation, and chemical advanced oxidation: a review. Sci Total Environ. (2009) 407:731–48. doi: 10.1016/j.scitotenv.2008.08.039, PMID: 18992918

[33] Liu ZH Lu GN Yin H Dang Z Rittmann B . Removal of natural estrogens and their conjugates in municipal wastewater treatment plants: a critical review. Environ Sci Technol. (2015) 49:5288–300. doi: 10.1021/acs.est.5b00399, PMID: 25844648

[34] Bowman RE Luine V Khandaker H Villafane JJ Frankfurt M . Adolescent bisphenol-A exposure decreases dendritic spine density: role of sex and age. Synapse. (2014) 68:498–507. doi: 10.1002/syn.21758, PMID: 24975924 PMC6112112

[35] Rezg R El-Fazaa S Gharbi N Mornagui B . Bisphenol A and human chronic diseases: current evidences, possible mechanisms, and future perspectives. Environ Int. (2014) 64:83–90. doi: 10.1016/j.envint.2013.12.007, PMID: 24382480

[36] Ayyavoo A . Gynecomastia. Indian J Pediatr. (2023) 90:1013–7. doi: 10.1007/s12098-023-04810-7, PMID: 37592101

[37] Ribeiro E Ladeira C Viegas S . EDCs mixtures: A stealthy hazard for human health? Toxics. (2017) 5. doi: 10.3390/toxics5010005, PMID: 29051438 PMC5606671

[38] Sharma K Lanzilotto A Yakubu J Therkelsen S Vöegel CD Du Toit T . Effect of essential oil components on the activity of steroidogenic cytochrome P450. Biomolecules. (2024) 14. doi: 10.3390/biom14020203, PMID: 38397440 PMC10887332

[39] Stevens S Mcpartland M Bartosova Z Skåland HS Völker J Wagner M . Plastic food packaging from five countries contains endocrine- and metabolism-disrupting chemicals. Environ Sci Technol. (2024) 58:4859–71. doi: 10.1021/acs.est.3c08250, PMID: 38441001 PMC10956434

[40] Plunk EC Richards SM . Endocrine-disrupting air pollutants and their effects on the hypothalamus-pituitary-gonadal axis. Int J Mol Sci. (2020) 21. doi: 10.3390/ijms21239191, PMID: 33276521 PMC7731392

[41] Casals-Casas C Desvergne B . Endocrine disruptors: from endocrine to metabolic disruption. Annu Rev Physiol. (2011) 73:135–62. doi: 10.1146/annurev-physiol-012110-142200, PMID: 21054169

[42] Geyer R Jambeck JR Law KL . Production, use, and fate of all plastics ever made. Sci Adv. (2017) 3:e1700782. doi: 10.1126/sciadv.1700782, PMID: 28776036 PMC5517107

[43] Landrigan PJ Raps H Cropper M Bald C Brunner M Canonizado EM . The minderoo-Monaco commission on plastics and human health. Ann Glob Health. (2023) 89:23. doi: 10.5334/aogh.4056, PMID: 36969097 PMC10038118

[44] Efsa Panel On Food Contact Materials E Processing A Lambre C Barat Baviera JM Bolognesi C Chesson A . Re-evaluation of the risks to public health related to the presence of bisphenol A (BPA) in foodstuffs. EFSA J. (2023) 21:e06857. doi: 10.2903/j.efsa.2023.6857, PMID: 37089179 PMC10113887

[45] Stackpoole SM Shoda ME Medalie L Stone WW . Pesticides in US Rivers: Regional differences in use, occurrence, and environmental toxicity, 2013 to 2017. Sci Total Environ. (2021) 787. doi: 10.1016/j.scitotenv.2021.147147, PMID: 33994194

[46] Zhang YJ Guo JL Xue JC Bai CL Guo Y . Phthalate metabolites: Characterization, toxicities, global distribution, and exposure assessment. Environ pollut. (2021) 291:118106. doi: 10.1016/j.envpol.2021.118106, PMID: 34520948

[47] Dai S Zhou Q Yang Y Zhang Y Zhang S Yao Y . Increasing contamination of polycyclic aromatic hydrocarbons in Chinese soils. J Environ Manage. (2024) 368:122268. doi: 10.1016/j.jenvman.2024.122268, PMID: 39178791

[48] Domínguez-López I Yago-Aragón M Salas-Huetos A Tresserra-Rimbau A Hurtado-Barroso S . Effects of dietary phytoestrogens on hormones throughout a human lifespan: A review. Nutrients. (2020) 12. doi: 10.3390/nu12082456, PMID: 32824177 PMC7468963

[49] Puche-Juarez M Toledano JM Moreno-Fernandez J Galvez-Ontiveros Y Rivas A Diaz-Castro J . The role of endocrine disrupting chemicals in gestation and pregnancy outcomes. Nutrients. (2023) 15. doi: 10.3390/nu15214657, PMID: 37960310 PMC10648368

[50] Rodprasert W Toppari J Virtanen HE . Endocrine disrupting chemicals and reproductive health in boys and men. Front Endocrinol (Lausanne). (2021) 12:706532. doi: 10.3389/fendo.2021.706532, PMID: 34690925 PMC8530230

[51] Eve L Fervers B Le Romancer M Etienne-Selloum N . Exposure to endocrine disrupting chemicals and risk of breast cancer. Int J Mol Sci. (2020) 21. doi: 10.3390/ijms21239139, PMID: 33266302 PMC7731339

[52] Pena-Corona SI Chavez-Corona JI Perez-Caltzontzin LE Vargas-Estrada D Mendoza-Rodriguez CA Ramos-Martinez E . Melatonin and vitamins as protectors against the reproductive toxicity of bisphenols: which is the most effective? A systematic review and meta-analysis. Int J Mol Sci. (2023) 24. doi: 10.3390/ijms241914930, PMID: 37834378 PMC10573514

[53] Xie R Wang X Xu Y Zhang L Ma M Wang Z . *In vitro* to *in vivo* extrapolation for predicting human equivalent dose of phenolic endocrine disrupting chemicals: PBTK model development, biological pathways, outcomes and performance. Sci Total Environ. (2023) 897:165271. doi: 10.1016/j.scitotenv.2023.165271, PMID: 37422235

[54] Shamhari A Abd Hamid Z Budin SB Shamsudin NJ Taib IS . Bisphenol A and its analogues deteriorate the hormones physiological function of the male reproductive system: A mini-review. Biomedicines. (2021) 9. doi: 10.3390/biomedicines9111744, PMID: 34829973 PMC8615890

[55] Peinado FM Iribarne-Duran LM Artacho-Cordon F . Human exposure to bisphenols, parabens, and benzophenones, and its relationship with the inflammatory response: A systematic review. Int J Mol Sci. (2023) 24. doi: 10.3390/ijms24087325, PMID: 37108488 PMC10139086

[56] Acconcia F Pallottini V Marino M . Molecular mechanisms of action of BPA. Dose Response. (2015) 13:1559325815610582. doi: 10.1177/1559325815610582, PMID: 26740804 PMC4679188

[57] Bolli A Galluzzo P Ascenzi P Del Pozzo G Manco I Vietri MT . Laccase treatment impairs bisphenol A-induced cancer cell proliferation affecting estrogen receptor alpha-dependent rapid signals. IUBMB Life. (2008) 60:843–52. doi: 10.1002/iub.130, PMID: 18767177

[58] Bolli A Bulzomi P Galluzzo P Acconcia F Marino M . Bisphenol A impairs estradiol-induced protective effects against DLD-1 colon cancer cell growth. IUBMB Life. (2010) 62:684–7. doi: 10.1002/iub.370, PMID: 20836126

[59] Varticovski L Stavreva DA Mcgowan A Raziuddin R Hager GL . Endocrine disruptors of sex hormone activities. Mol Cell Endocrinol. (2022) 539:111415. doi: 10.1016/j.mce.2021.111415, PMID: 34339825 PMC8762672

[60] La Merrill MA Vandenberg LN Smith MT Goodson W Browne P Patisaul HB . Consensus on the key characteristics of endocrine-disrupting chemicals as a basis for hazard identification. Nat Rev Endocrinol. (2020) 16:45–57. doi: 10.1038/s41574-019-0273-8, PMID: 31719706 PMC6902641

[61] Shafei A Ramzy MM Hegazy AI Husseny AK El-Hadary UG Taha MM . The molecular mechanisms of action of the endocrine disrupting chemical bisphenol A in the development of cancer. Gene. (2018) 647:235–43. doi: 10.1016/j.gene.2018.01.016, PMID: 29317319

[62] Li DK Zhou Z Miao M He Y Wang J Ferber J . Urine bisphenol-A (BPA) level in relation to semen quality. Fertil Steril. (2011) 95:625–630 e621-624. doi: 10.1016/j.fertnstert.2010.09.026, PMID: 21035116

[63] Hunt PA Lawson C Gieske M Murdoch B Smith H Marre A . Bisphenol A alters early oogenesis and follicle formation in the fetal ovary of the rhesus monkey. Proc Natl Acad Sci. (2012) 109:17525–30. doi: 10.1073/pnas.1207854109, PMID: 23012422 PMC3491481

[64] Hunt PA Koehler KE Susiarjo M Hodges CA Ilagan A Voigt RC . Bisphenol a exposure causes meiotic aneuploidy in the female mouse. Curr Biol. (2003) 13:546–53. doi: 10.1016/S0960-9822(03)00189-1, PMID: 12676084

[65] Jurewicz J Majewska J Berg A Owczarek K Zajdel R Kaleta D . Serum bisphenol A analogues in women diagnosed with the polycystic ovary syndrome - is there an association? Environ pollut. (2021) 272:115962. doi: 10.1016/j.envpol.2020.115962, PMID: 33223334

[66] Rochester JR Bolden AL . Bisphenol S and F: A systematic review and comparison of the hormonal activity of bisphenol A substitutes. Environ Health Perspect. (2015) 123:643–50. doi: 10.1289/ehp.1408989, PMID: 25775505 PMC4492270

[67] Yokota H Iwano H Endo M Kobayashi T Inoue H Ikushiro S . Glucuronidation of the environmental oestrogen bisphenol A by an isoform of UDP-glucuronosyltransferase, UGT2B1, in the rat liver. [J]. Biochem J, (1999) 340:405–9., PMID: 10333482 PMC1220264

[68] Phrakonkham P Viengchareun S Belloir C Lombes M Artur Y Canivenc-Lavier MC . Dietary xenoestrogens differentially impair 3T3-L1 preadipocyte differentiation and persistently affect leptin synthesis. J Steroid Biochem Mol Biol. (2008) 110:95–103. doi: 10.1016/j.jsbmb.2008.02.006, PMID: 18359623

[69] Somm E Schwitzgebel VM Toulotte A Cederroth CR Combescure C Nef S . Perinatal exposure to bisphenol a alters early adipogenesis in the rat. Environ Health Perspect. (2009) 117:1549–55. doi: 10.1289/ehp.11342, PMID: 20019905 PMC2790509

[70] Wada K Sakamoto H Nishikawa K Sakuma S Nakajima A Fujimoto Y . Life style-related diseases of the digestive system: endocrine disruptors stimulate lipid accumulation in target cells related to metabolic syndrome. J Pharmacol Sci. (2007) 105:133–7. doi: 10.1254/jphs.FM0070034, PMID: 17928741

[71] Alonso-Magdalena P Morimoto S Ripoll C Fuentes E Nadal A . The estrogenic effect of bisphenol A disrupts pancreatic beta-cell function *in vivo* and induces insulin resistance. Environ Health Perspect. (2006) 114:106–12. doi: 10.1289/ehp.8451, PMID: 16393666 PMC1332664

[72] Jayashree S Indumathi D Akilavalli N Sathish S Selvaraj J Balasubramanian K . Effect of Bisphenol-A on insulin signal transduction and glucose oxidation in liver of adult male albino rat. Environ Toxicol Pharmacol. (2013) 35:300–10. doi: 10.1016/j.etap.2012.12.016, PMID: 23376180

[73] Vita R Capodicasa G Di Bari F Amadeo G Stagno F Benvenga S . Biochemical features of eugonadal patients with idiopathic gynaecomastia: A retrospective cross-sectional study. Andrologia. (2021) 53:e13962. doi: 10.1111/and.13962, PMID: 33411368

[74] Murata M Kang JH . Bisphenol A (BPA) and cell signaling pathways. Biotechnol Adv. (2018) 36:311–27. doi: 10.1016/j.biotechadv.2017.12.002, PMID: 29229539

[75] Lacorte S Latorre A Guillamon M Barcelo D . Nonylphenol, octyphenol, and bisphenol A in groundwaters as a result of agronomic practices. ScientificWorldJournal. (2002) 2:1095–100. doi: 10.1100/tsw.2002.219, PMID: 12805966 PMC6009321

[76] Mao Z Zheng XF Zhang YQ Tao XX Li Y Wang W . Occurrence and biodegradation of nonylphenol in the environment. Int J Mol Sci. (2012) 13:491–505. doi: 10.3390/ijms13010491, PMID: 22312266 PMC3269700

[77] Wang S Wu W Liu F Yin S Bao Z Liu H . Spatial distribution and migration of nonylphenol in groundwater following long-term wastewater irrigation. J Contam Hydrol. (2015) 177-178:85–92. doi: 10.1016/j.jconhyd.2015.03.013, PMID: 25886245

[78] Lee HJ Chattopadhyay S Gong EY Ahn RS Lee K . Antiandrogenic effects of bisphenol A and nonylphenol on the function of androgen receptor. Toxicol Sci. (2003) 75:40–6. doi: 10.1093/toxsci/kfg150, PMID: 12805653

[79] Dimitrakakis C Bondy C . Androgens and the breast. Breast Cancer Res. (2009) 11:212. doi: 10.1186/bcr2413, PMID: 19889198 PMC2790857

[80] Tao S Yao Z Li H Wang Y Qiao X Yu Y . Exposure to 4-nonylphenol compromises Leydig cell development in pubertal male mice. Ecotoxicol Environ Saf. (2023) 266:115612. doi: 10.1016/j.ecoenv.2023.115612, PMID: 37866035

[81] Chen S Shi Z Zhang Q . A physiologically based pharmacokinetic model of diethyl phthalates in humans. Environ pollut. (2024) 340:122849. doi: 10.1016/j.envpol.2023.122849, PMID: 37926418 PMC10841618

[82] Mariana M Feiteiro J Verde I Cairrao E . The effects of phthalates in the cardiovascular and reproductive systems: A review. Environ Int. (2016) 94:758–76. doi: 10.1016/j.envint.2016.07.004, PMID: 27424259

[83] Morgenstern R Whyatt RM Insel BJ Calafat AM Liu X Rauh VA . Phthalates and thyroid function in preschool age children: Sex specific associations. Environ Int. (2017) 106:11–8. doi: 10.1016/j.envint.2017.05.007, PMID: 28554096 PMC5533628

[84] Frederiksen H Skakkebaek NE Andersson AM . Metabolism of phthalates in humans. Mol Nutr Food Res. (2007) 51:899–911. doi: 10.1002/mnfr.200600243, PMID: 17604388

[85] Shukla R Kannan A Laws MJ Wagoner Johnson A Flaws JA Bagchi MK . Exposure to phthalates enhances estrogen and beta-catenin signaling pathways, leading to endometrial hyperplasia in mice. Toxicol Sci. (2025) 206:58–67. doi: 10.1093/toxsci/kfaf062, PMID: 40323316 PMC12198673

[86] Dairkee SH Moore DH Luciani MG Anderle N Gerona R Ky K . Reduction of daily-use parabens and phthalates reverses accumulation of cancer-associated phenotypes within disease-free breast tissue of study subjects. Chemosphere. (2023) 322:138014. doi: 10.1016/j.chemosphere.2023.138014, PMID: 36746253

[87] Street ME Angelini S Bernasconi S Burgio E Cassio A Catellani C . Current knowledge on endocrine disrupting chemicals (EDCs) from animal biology to humans, from pregnancy to adulthood: highlights from a national italian meeting. Int J Mol Sci. (2018) 19. doi: 10.3390/ijms19061647, PMID: 29865233 PMC6032228

[88] Zhang J Gu W Zhai S Liu Y Yang C Xiao L . Phthalate metabolites and sex steroid hormones in relation to obesity in US adults: NHANES 2013–2016. Front Endocrinol (Lausanne). (2024) 15:1340664. doi: 10.3389/fendo.2024.1340664, PMID: 38524635 PMC10957739

[89] Shah SS Kanani EAM Kharat SK Shah PS Shah RM . Evaluation of the incidence of low testosterone levels in young male adults with moderate to severe obesity-single-centre study from India. Obes Surg. (2024) 34:836–40. doi: 10.1007/s11695-024-07075-x, PMID: 38282174

[90] Billa E Kanakis GA Goulis DG . Imaging in gynecomastia. Andrology. (2021) 9:1444–56. doi: 10.1111/andr.13051, PMID: 34033252

[91] Barbosa Jr. F Rocha BA Souza MCO Bocato MZ Azevedo LF Adeyemi JA . Polycyclic aromatic hydrocarbons (PAHs): Updated aspects of their determination, kinetics in the human body, and toxicity. J Toxicol Environ Health B Crit Rev. (2023) 26:28–65. doi: 10.1080/10937404.2022.2164390, PMID: 36617662

[92] Bekki K Toriba A Tang N Kameda T Hayakawa K . Biological effects of polycyclic aromatic hydrocarbon derivatives. J UOEH. (2013) 35:17–24. doi: 10.7888/juoeh.35.17, PMID: 23475020

[93] Kummer V Maskova J Zraly Z Neca J Simeckova P Vondracek J . Estrogenic activity of environmental polycyclic aromatic hydrocarbons in uterus of immature Wistar rats. Toxicol Lett. (2008) 180:212–21. doi: 10.1016/j.toxlet.2008.06.862, PMID: 18634860

[94] Bukowska B Sicinska P . Influence of benzo(a)pyrene on different epigenetic processes. Int J Mol Sci. (2021) 22. doi: 10.3390/ijms222413453, PMID: 34948252 PMC8707600

[95] Romagnolo DF Papoutsis AJ Laukaitis C Selmin OI . Constitutive expression of AhR and BRCA-1 promoter CpG hypermethylation as biomarkers of ERalpha-negative breast tumorigenesis. BMC Cancer. (2015) 15:1026. doi: 10.1186/s12885-015-2044-9, PMID: 26715507 PMC4696163

[96] Gearhart-Serna LM Davis JB Jolly MK Jayasundara N Sauer SJ Di Giulio RT . A polycyclic aromatic hydrocarbon-enriched environmental chemical mixture enhances AhR, antiapoptotic signaling and a proliferative phenotype in breast cancer cells. Carcinogenesis. (2020) 41:1648–59. doi: 10.1093/carcin/bgaa047, PMID: 32747956 PMC7791619

[97] Sahay D Lloyd SE Rivera JA Jezioro J Mcdonald JD Pitiranggon M . Prenatal polycyclic aromatic hydrocarbons, altered ERalpha pathway-related methylation and expression, and mammary epithelial cell proliferation in offspring and grandoffspring adult mice. Environ Res. (2021) 196:110961. doi: 10.1016/j.envres.2021.110961, PMID: 33675803 PMC8119355

[98] Andre S SP N Silva F Henrique R Felix A Jeronimo C . Analysis of epigenetic alterations in homologous recombination DNA repair genes in male breast cancer. Int J Mol Sci. (2020) 21. doi: 10.3390/ijms21082715, PMID: 32295201 PMC7215617

[99] Ling J Yan Z Liu X Men S Wei C Wang Z . Health risk assessment and development of human health ambient water quality criteria for PCBs in Taihu Basin, China. Sci Total Environ. (2024) 920:170669. doi: 10.1016/j.scitotenv.2024.170669, PMID: 38316297

[100] Zani C Toninelli G Filisetti B Donato F . Polychlorinated biphenyls and cancer: an epidemiological assessment. J Environ Sci Health C Environ Carcinog Ecotoxicol Rev. (2013) 31:99–144. doi: 10.1080/10590501.2013.782174, PMID: 23672403

[101] Lerro CC Jones RR Langseth H Grimsrud TK Engel LS Sjodin A . A nested case-control study of polychlorinated biphenyls, organochlorine pesticides, and thyroid cancer in the Janus Serum Bank cohort. Environ Res. (2018) 165:125–32. doi: 10.1016/j.envres.2018.04.012, PMID: 29698872 PMC5999553

[102] Cheng Z Zhang X Bassig B Hauser R Holford TR Zheng E . Serum polychlorinated biphenyl (PCB) levels and risk of testicular germ cell tumors: A population-based case-control study in Connecticut and Massachusetts. Environ pollut. (2021) 273:116458. doi: 10.1016/j.envpol.2021.116458, PMID: 33482463

[103] Lim JE Nam C Yang J Rha KH Lim KM Jee SH . Serum persistent organic pollutants (POPs) and prostate cancer risk: A case-cohort study. Int J Hyg Environ Health. (2017) 220:849–56. doi: 10.1016/j.ijheh.2017.03.014, PMID: 28420543

[104] Parada Jr. H Sun X Tse CK Engel LS Hoh E Olshan AF . Plasma levels of polychlorinated biphenyls (PCBs) and breast cancer mortality: The Carolina Breast Cancer Study. Int J Hyg Environ Health. (2020) 227:113522. doi: 10.1016/j.ijheh.2020.113522, PMID: 32276222 PMC7387141

[105] Tam N Lai KP Kong RYC . Comparative transcriptomic analysis reveals reproductive impairments caused by PCBs and OH-PCBs through the dysregulation of ER and AR signaling. Sci Total Environ. (2022) 802:149913. doi: 10.1016/j.scitotenv.2021.149913, PMID: 34474298

[106] Carrera ARM Eleazar EG Caparanga AR Tayo LL . Theoretical studies on the quantitative structure-toxicity relationship of polychlorinated biphenyl congeners reveal high affinity binding to multiple human nuclear receptors. Toxics. (2024) 12. doi: 10.3390/toxics12010049, PMID: 38251005 PMC10821279

[107] Trasande L Urbina EM Khoder M Alghamdi M Shabaj I Alam MS . Polycyclic aromatic hydrocarbons, brachial artery distensibility and blood pressure among children residing near an oil refinery. Environ Res. (2015) 136:133–40. doi: 10.1016/j.envres.2014.08.038, PMID: 25460629 PMC5274701

[108] Atteia HH . A combination of silymarin and garlic extract enhances thyroid hormone activation and body metabolism in orally intoxicated male rats with atrazine: Impact on hepatic iodothyronine deiodinase type 1. Pestic Biochem Physiol. (2024) 199:105801. doi: 10.1016/j.pestbp.2024.105801, PMID: 38458692

[109] Zhao M Ma J Li M Zhang Y Jiang B Zhao X . Cytochrome P450 enzymes and drug metabolism in humans. Int J Mol Sci. (2021) 22. doi: 10.3390/ijms222312808, PMID: 34884615 PMC8657965

[110] Mcmullin TS Brzezicki JM Cranmer BK Tessari JD Andersen ME . Pharmacokinetic modeling of disposition and time-course studies with [14C]atrazine. J Toxicol Environ Health A. (2003) 66:941–64. doi: 10.1080/15287390306454, PMID: 12825238

[111] Abel EL Opp SM Verlinde CL Bammler TK Eaton DL . Characterization of atrazine biotransformation by human and murine glutathione S-transferases. Toxicol Sci. (2004) 80:230–8. doi: 10.1093/toxsci/kfh152, PMID: 15115887

[112] Kaprara A Huhtaniemi IT . The hypothalamus-pituitary-gonad axis: Tales of mice and men. Metabolism. (2018) 86:3–17. doi: 10.1016/j.metabol.2017.11.018, PMID: 29223677

[113] Rotimi DE Ojo OA Adeyemi OS . Atrazine exposure caused oxidative stress in male rats and inhibited brain-pituitary-testicular functions. J Biochem Mol Toxicol. (2024) 38:e23579. doi: 10.1002/jbt.23579, PMID: 37926918

[114] Kolaitis ND Finger BJ Merriner DJ Nguyen J Houston BJ O'bryan MK . Impact of chronic multi-generational exposure to an environmentally relevant atrazine concentration on testicular development and function in mice. Cells. (2023) 12. doi: 10.3390/cells12040648, PMID: 36831314 PMC9954248

[115] Harper AP Finger BJ Green MP . Chronic atrazine exposure beginning prenatally impacts liver function and sperm concentration with multi-generational consequences in mice. Front Endocrinol (Lausanne). (2020) 11:580124. doi: 10.3389/fendo.2020.580124, PMID: 33324343 PMC7726345

[116] Liu C Akbariyeh S Bartelt-Hunt S Li Y . Impacts of future climate variability on atrazine accumulation and transport in corn production areas in the midwestern United States. Environ Sci Technol. (2022) 56:7873–82. doi: 10.1021/acs.est.2c00029, PMID: 35649150

[117] Kaur R Choudhary D Bali S Bandral SS Singh V Ahmad MA . Pesticides: An alarming detrimental to health and environment. Sci Total Environ. (2024) 915:170113. doi: 10.1016/j.scitotenv.2024.170113, PMID: 38232846

[118] Yang C Song G Lim W . Effects of endocrine disrupting chemicals in pigs. Environ pollut. (2020) 263:114505. doi: 10.1016/j.envpol.2020.114505, PMID: 32268228

[119] Jugan J Lind PM Salihovic S Stubleski J Karrman A Lind L . The associations between p,p’-DDE levels and plasma levels of lipoproteins and their subclasses in an elderly population determined by analysis of lipoprotein content. Lipids Health Dis. (2020) 19:249. doi: 10.1186/s12944-020-01417-1, PMID: 33287856 PMC7722417

[120] Yaglova NV Tsomartova DA Yaglov VV . Differences in adrenal steroid hormones production in pubertal rats exposed to low doses of endocrine disruptor DDT during prenatal and postnatal development. BioMed Khim. (2017) 63:306–11. doi: 10.18097/PBMC20176304306, PMID: 28862600

[121] Wang L Qie Y Yang Y Zhao Q . Binding and activation of estrogen-related receptor gamma: A novel molecular mechanism for the estrogenic disruption effects of DDT and its metabolites. Environ Sci Technol. (2022) 56:12358–67. doi: 10.1021/acs.est.1c08624, PMID: 35947429

[122] Kalyan S . Prepubertal gynecomastia linked to lavender and tea tree oils. N Engl J Med. (2007) 356:2542., PMID: 17575592

[123] Hareng L Kolle SN Gomes C Schneider S Wahl M . Critical assessment of the endocrine potential of Linalool and Linalyl acetate: proactive testing strategy assessing estrogenic and androgenic activity of Lavender oil main components. Arch Toxicol. (2023) 98:347–61. doi: 10.1007/s00204-023-03623-z, PMID: 37906319 PMC10761525

