# Supplementary material for: Potential association between endocrine disrupting chemicals (EDCs) and gynecomastia: a systematic review based on partial experimental evidenceendocrine-disrupting chemicals
Source: Front Endocrinol (Lausanne). 2026 Feb 3;17:1734644. doi: 10.3389/fendo.2026.1734644 (PMC12909171; doi:10.3389/fendo.2026.1734644)
Supplement: Supplementary file 1 [file Supplementaryfile1.docx]

**Identification of studies via databases and registers**

Records removed *before screening*:

Duplicate records removed (n = 260)

Records marked as ineligible by automation tools (n =0 )

Records removed for other reasons (n =0 )

Records identified from

PubMed (n =1500)

**Identification**

Records screened

(n = 1240)

Records excluded**

(n =300 )

Reports sought for retrieval

(n =1420 )

Reports not retrieved

(n =130 )

**Screening**

Reports assessed for eligibility

(n = 1290)

Reports excluded:

Studies involving female subjects (n =220 )

Purely toxicological research that fails to establish a clear association between EDCs and male breast development (n =685 )

Duplicated publications or studies of low quality(n =260 )

Studies included in review

(n = 125)

**Included**
